# Utilizing Yin-Yang approach to reinforce fuzzy front-end activities and manufacturing startups’ growth performance

**DOI:** 10.1371/journal.pone.0306779

**Published:** 2024-10-23

**Authors:** Jiaxu Huang, Haiqing Hu

**Affiliations:** 1 School of Economics and Management, Xi’an Aeronautical Institute, Xi’an, China; 2 School of Economics and Management, Xi’an University of Technology, Xi’an, China; University of Nottingham Ningbo China, CHINA

## Abstract

Fuzzy front-end is the research frontier of manufacturing industry. This study investigates the relationship between market-oriented FFE activities and manufacturing startup growth performance by adopting an open innovative ancient Chinese Taoism Yin-Yang approach under this post-pandemic circumstance. This study also examines the moderating behavioral effect of Chinese Zen-originated CEO mindfulness between market-oriented FFE activities and manufacturing startup growth performance. Data for this study were gathered from 343 Chinese manufacturing startups’ CEOs across different manufacturing industries, including both high-tech and conventional manufacturing industries. The collected data were analyzed by using structural equation modeling and Bootstrap method. The quantitative analysis results show that most market-oriented FFE activities have positive effects on manufacturing startup growth performance and the Chinese Zen-originated CEO mindfulness positively moderates the relationship between most FFE activities and startup growth performance.

## 1. Introduction

The existing influential studies on manufacturing firms’ fuzzy front-end (FFE) innovation activities mostly concern the new product development (NPD) stage of firms’ growth process [1–7], stating that FFE activities are the momentous initial phase of NPD and that these activities prompt NPD success [8–12]. However, the COVID-19 pandemic has reshaped the world of business, disrupting firms’ sales activities and international trade and supply chains, especially for small startups [13,14]. How did startups survive the extremely uncertain market during this age characterized by volatility, uncertainty, complexity and ambiguity (VUCA) to maintain economic growth? We affirm that it is crucial for startups to rethink the way that they construct new product concepts both creatively and innovatively during the FFE stage to alleviate the increasing uncertainties both inside and outside firms and stimulate firm-level growth in the long run since both creativity and innovation have major impacts on firm growth performance [15–18]. However, there is a lack of research on this matter in terms of how FFE activities influence firm growth performance from a broad perspective. Although Markham [11] summarized five FFE activities and their impacts on product performance through the mediator of FFE performance from a managerial perspective, the specific actions needed to design an early product prototype during the FFE stage remain debatable, as researchers have not reached a consensus on this issue. However, integrating marketing strategy with product design during the upstream FFE phase of NPD helps firms to concisely identify market demand in advance for generating effective outcomes [19]. Therefore, in this research, we define FFE activities from a downstream market-oriented perspective, investigating the relationship between FFE activities and manufacturing startup growth performance.

According to enterprise life cycle theory, startup companies have more growth potential than mature firms. On the other hand, startups are generally financially fragile and exhibit an informal work ethic and an ambiguous hierarchy [20]. A lack of top management engagement makes startup’s FFE process relatively uncertain [7], which suggests that experienced startup CEO leadership could be useful in determining startups’ direction under the cloud of uncertainties they face. The FFE process requires mindful leadership to guide product completion procedures for good consumer acceptance, as FFE activities are usually ambiguous and unclear [9]. Moreover, effective leadership is the engine of the FFE process that initiates FFE activities [21]. Scholarly evidence suggests that mindfulness, a practice that originated in ancient Chinese Zen Buddhism, helps CEOs make better decisions under extreme pressure during the COVID-19 pandemic [22]. Hence, in this research, we test the moderating effect of CEOs’ mindfulness practices during the pandemic on the relationship between market-oriented FFE activities and startups’ growth performance based on evidence from Chinese startups from a holistic point of view.

The COVID-19 pandemic caused extensive economic damage, and we believe that open innovation Taoism yin-yang adoption addresses FFE uncertainties well by processing information and establishing FFE activities to reduce FFE uncertainties as it provides an alternative angle to rethink the current business world; this is because an off-boundary environment helps transfer as much market information as possible since market uncertainty varies from day to day during the pandemic [5–7,23,24]. However, there is a lack of research on this matter. In our opinion, the Chinese harmonious Yin-Yang approach enhances FFE innovation because it is, unlike the frequently-used western approach, a soft power to rather digest the economic uncertainties and frictions [25]. Therefore, in this study, we first specifically categorized FFE activities based on market demand and new product commercialization, prioritizing the relationship between FFE activities and startup growth performance with a Taoism yin-yang approach [26,27]; second, we add a micro view to FFE research, evaluating the moderating effects of CEOs’ Zen Buddhism mindfulness in facilitating a successful impact of the relationship between FFE activities on startup growth performance. Hence, the scope of this study is to utilize ancient Chinese philosophy to reinforce the traditional western thinking of FFE and manufacturing practices, adding a new aspect to solve innovation and entrepreneurial problems that the business world is facing currently. To sum up, we hope to solve three research problems in this research:

RQ1: How does Chinese Yin-Yang approach be compatible with exiting FFE literature? Is this approach better in dealing with FFE uncertainties? What is the rationale behind it?
RQ2: Do Market-oriented FFE activities truly increase manufacturing startups’ growth performance?
RQ: Facing the current economic uncertainties, does Zen Buddhism based CEO mindfulness positively moderate the relationship between FFE activities and startup growth performance?

This paper is organized as follows. First, we illustrate our research paper’s theoretical background, developing a set of hypotheses that corresponds to our theoretical conceptual model. We further conduct a formal survey among manufacturing startups to test proposed hypotheses. Finally, we provide our findings and both the theoretical and managerial implications of our study, outlining its limitations and future research opportunities.

## 2. Theoretical foundation

### 2.1. The open innovation model of FFE based on a Yin-Yang approach

The FFE innovation of a manufacturing startup is initiated with the hope of producing as output a primary product prototype that can be substantially replicated and successfully launched into the market during subsequent stages of the NPD process [8,28]. The FFE process usually starts when an opportunity is first recognized and ends when the corresponding firm has decided to either develop or terminate the product [8,9,12]. Therefore, FFE activities are the most fundamental initiatives for a startup aiming to set up a product’s market commercialization blueprint by decreasing NPD duration and costs.

**Fig 1:**
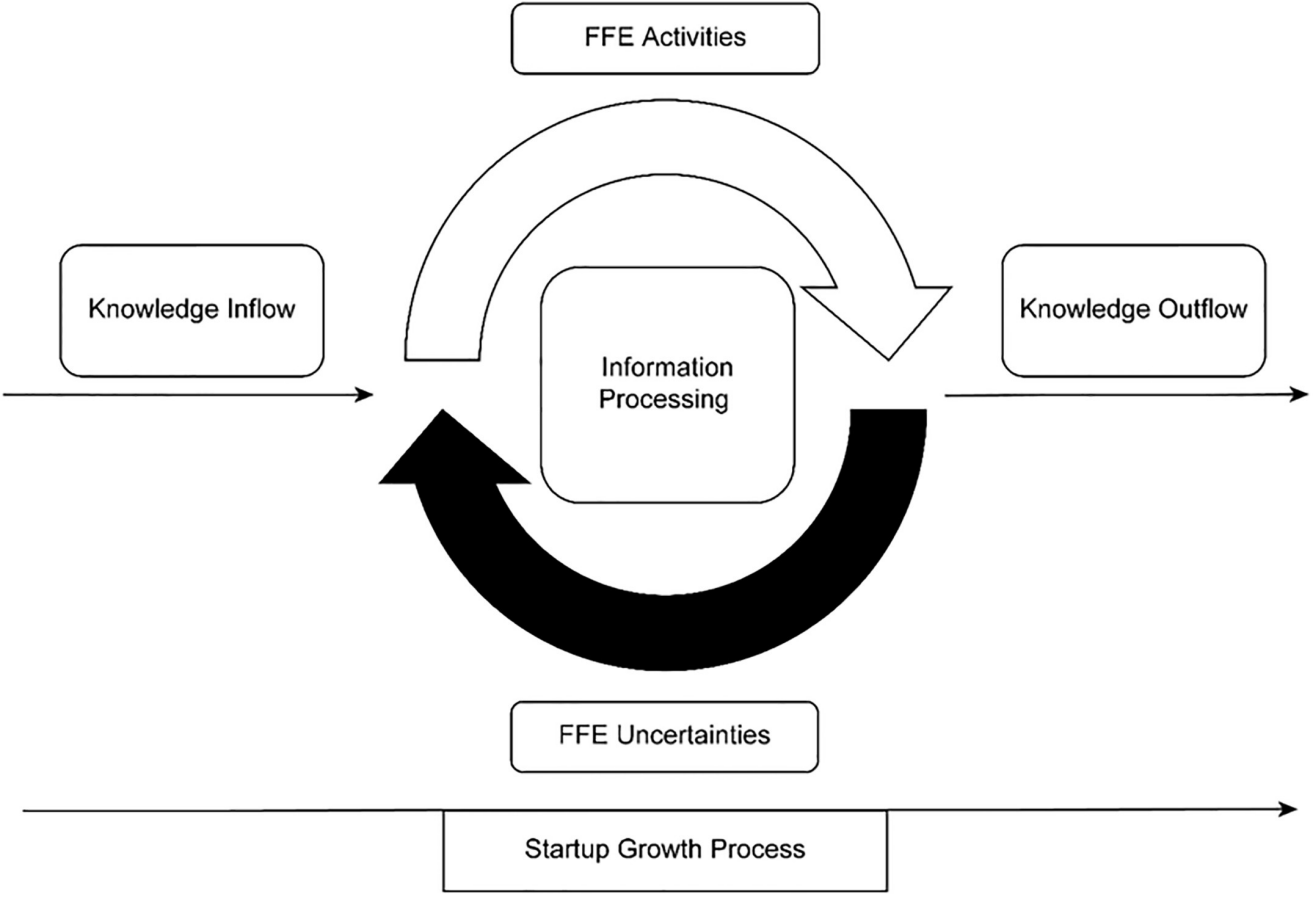
The Yin-Yang approach to open innovation FFE model. The wheel-like FFE model starts to roll either forward or backward when a new product is being constructed conceptually during the FFE stage. It stops rolling when the new product is approved for volume production.

As the initial phase of new product innovation, FFE innovation is filled with uncertainty from the market (customers and competitors) and rapidly developing technologies [29,30]. Hence, firms must process information from both their internal environments and their external environments to accurately execute FFE activities, reduce uncertainties and create market-proven products [5–8,31]. Thus, an open-boundary environment stimulates startups’ organizational learning, accelerating their FFE information processing and thus enabling better FFE activity performance to reduce uncertainties stochastically [32] depending on the quality and speed of external information [2]. Firms with better absorptive capacity for external knowledge have better overall performance [33]; moreover, a higher level of FFE openness competence can draw different positive factors from the external environment to decrease uncertainties, making FFE innovation less chaotic [34]. Therefore, in this research, we apply the open innovation theory proposed by [23,24] to the FFE innovation process. Chesbrough [24] defined open innovation as “purposeful inflows and outflows of knowledge to accelerate innovation internally while also expanding the markets for the external use of innovation”. Through this open boundary knowledge flow process, inflows of knowledge enhance information processing to encourage more efficient innovative FFE activities for uncertainty reduction, whereas outflows of knowledge help build an ecosystem where the focal firm can attract more allies by sharing knowledge with them; this makes the firm an industry leader. Additionally, based on open innovation theory, we applied a Chinese Taoism Yin-Yang balanced approach to this open innovation FFE model [25], reforming the mechanism of the FFE activity uncertainty reduction process depicted by other scholarly evidence [5–7,31] via modest dualism so that harmony is the goal of managing the conflict between FFE activities and FFE uncertainties [35]. As FFE uncertainties can be reduced but never eliminated, we construct our proposed FFE model by applying the Taoism Yin-Yang approach: FFE activities and uncertainties interact with each other in an open environment as knowledge flows in and out to prevent fierce conflicts as if two opposing Yin and Yang elements were circulating with each other until reaching a dynamic equilibrium [25], as shown in Fig 1.

Cropley and Oppert [36] also argued that a dynamic process stimulates creativity, making the management of FFE more effective. In this FFE uncertainty reduction progress, a dualist Yin-Yang approach resolves FFE uncertainties in such a way that FFE uncertainties are treated not as an obstacle but rather as a perpetual feedback stimulus that inspires creativity since FFE creativity is constantly being structured and evaluated to refine the best product for uncertain markets; thus, it improves a firm’s innovation ability [37]. In eastern Asian countries, especially China, where Taoism originated, such Taoism Yin-Yang thinking would include the idea that two opposite factors can balance by smoothing with each other instead of confronting one another [38,39].

As Dodgson [40] claimed, organizational learning is an effective way to establish organizational efficiency by assembling knowledge from both individuals and organizations inside and outside the focal organization. In this study, we propose that it is critical to ascertain manufacturing startups’ strategies during the FFE stage for the upcoming commercialization phase when products are being sold in the market. Therefore, this Yin-Yang approach to the open innovation FFE model involves a learning process that helps startups learn both explicit and tacit knowledge as organizations for a minimally uncertain market offering. Consequently, we propose that FFE activities are not intended to eliminate uncertainties but rather to enable coexistence with them creatively in an interactive way to create a lasting open and sustainable innovation environment and facilitate adjustments to changing future circumstances at any moment. This paradox mindset of FFE implementation has been suggested by scholars to drive innovation forward when the freedom of innovation and the regulation of uncertainties concomitantly interact with one another [1]. Consequently, this open innovation model of FFE (Fig 1) can be seen as a simulative application of Norbert Wiener’s cybernetics theory.

### 2.2. A classification of downstream market-oriented FFE activities

Although the establishment of market and technology analysis is required by FFE innovation [8], our study adapts the market (demand)-pull FFE innovation [26,27] approach based on our open innovation FFE model presented based on the preceding literature. A market-pull strategy requires less time and cost for product development to prevent market uncertainty than technology-push FFE innovation because “outside-in” market-pull FFE innovation focuses on the open market environment prior to a firm’s own technology advancement, whereas technology-push FFE innovation, which is “inside-out”, limits a firm to starting with technology advancement inside the firm rather than watching out for market needs [26,41]. In our opinion, the FFE of NPD is a business process rather than scientific research; thus, the market orientation should be ahead of technological progress, and technology should ultimately serve market demand because the goal of a final product is to satisfy customers’ and stakeholders’ core demands and requirements. Especially during the post-COVID-19 pandemic concussion, adopting a market perspective in the context of FFE can help startups execute FFE activities and other firm-level actions based on a more versatile, lean management approach that is highly responsive to market change, alerting these firms to market uncertainty in a timely manner to avoid unnecessary work that is costly in terms of both time and money, thus maintaining a long-term relationship with stakeholders to earn sustainable profits.

While most significant innovation decisions are made during the FFE and product commercialization phases [42], both the findings of Murphy and Kumar [12] and those of Sandmeier et al. [43] have included the assessment of market commercialization as a key component of FFE. This approach highlights the importance of integrating downstream market commercialization as a key solution that provides market information and knowledge to upstream FFE innovation processes ahead of time concerning customer needs, prototype feedback and competitors’ reactions [44]. The technology side of manufacturing ensures the quality of a product, whereas FFE activities with market commercialization concerns can help startups tell a good story about a product in advance, increasing the product’s market share. Beverland et al. [19] also showed that integrating downstream marketing strategy into the upstream product design process of a firm’s value chain significantly enhances NPD success, improving firm growth performance. Through a series of market-guided FFE activities, startups can build an open innovation ecosystem that allows them to collaborate with different stakeholders and thus understand the market in advance to prevent unnecessary product failures during the downstream commercialization stage; this leads to superior organizational performance and improved NPD performance from a resource-based perspective [2]. On the other hand, careful attention to market commercialization in FFE activity development also allows firms to acquire resources from the market for downstream use, accelerating the FFE process. Moreover, accelerating FFE innovation is the key that enables startups to overtake imitators and level up their growth based on market needs, especially in the rapidly changing market of the COVID-19 era [9,10,45].

Based on an analysis of previous studies, the classification of FFE activities are disputed in different ways. Cooper and Kleinsehmidt [46] listed idea generation, product assessment and concept definition; Murphy and Kumar [12] highlighted idea generation, product definition and project evaluation as FFE activities; Khurana and Rosenthal [8] considered opportunity identification and assessment, idea generation, product strategy formulation and communication, product definition, project planning and executive reviews as FFE activities intended to determine whether products should be launched in the market; Nobelius and Trygg [47] proposed that mission statements, concept generation, concept screening, concept definition, business analysis and project planning make up FFE activities; and Koen et al. [21] recognized opportunity identification, opportunity analysis, idea generation, idea selection, concept definition and concept development as FFE activities.

In our study on open innovative market-oriented FFE activities, we propose that the external stakeholders of a manufacturing firm’s vertical value chain (customers and suppliers) are also important FFE actors needed to maintain market alertness during the FFE stage [48], as the findings of Koen et al. [21], Zhang and Doll [6], Zhang et al. [7], Murphy and Kumar [12], Sandmeier et al. [43], and Kim and Wilemon [9,10] all emphasized the importance of FFE external involvement. Activities from the external environment during the product design phase, such as customer involvement and supplier involvement, have been proven to be key measures to impact of downstream firm performance on a firm’s vertical value chain because vertical stakeholders (customers and suppliers) have positive impact on feasibility and legitimacy of a product later during the product commercialization phase [21,49,50]. Moreover, Tran et al. [51] illustrate that interactions with customers have major impacts on FFE innovation by comparing FFE with a city orchestra’s creative process. Furthermore, the findings of Murphy and Kumar [12] and Sandmeier et al. [43] proved that prototyping is an important FFE activity. Christiansen and Gasparin [28] showed that a feasible prototype is the final objective of an FFE process. In our opinion, FFE prototyping is the culmination of all other conceptual FFE activities. We view FFE prototyping as the summary of all FFE opportunities into an actual object. FFE innovation is complete when a final prototype is ready to be evaluated in terms of whether the next NPD steps will be undertaken. Therefore, we divide downstream market-oriented concurrent FFE activities into four dimensions: FFE opportunity recognition, FFE concept generation, FFE vertical external involvement (customers and suppliers) and FFE prototyping.

### 2.3. CEO mindfulness

The CEO is the core strategist of a startup manufacturer and must be generally knowledgeable in terms of leading the FFE process [9] because he or she is usually deeply involved with the firm’s growth process; this is because startup teams are generally small and relatively nonhierachical [20]. Moreover, the findings of Markham [11], Koen et al. [21], and Kurkkio [52] revealed that the involvement of top management positively affects a firm’s FFE performance and is crucial for product performance. During the post-COVID-19 VUCA era, startup CEOs need much energy to find the unchanging inner power within the ever-changing market to guide their startups in the right direction for survival; indeed, Koen et al. [21] emphasized that leadership is the engine of the whole FFE process.

However, in our opinion, startup CEOs should leave the execution of certain FFE innovation activities to FFE teams so that they can mindfully evaluate their inner worlds through introspection and thus make unhindered decisions regarding the orientation of their long-term direction. Although information processing is vital in terms of enabling FFE teams to organize FFE activities and thus decrease uncertainties [5–7], an excess of information slows FFE and misleads CEOs’ decisions related to guiding their startups’ future direction. Therefore, on the other hand, startup CEOs should give directions based on their mindful inner intuition from their tacit knowledge reserves; indeed, Kim and Wilemon [10] suggested that FFE managers forecast the evolution of markets and technology. From this point of view, a startup manufacturer is in need of a flexible and supportive CEO that mindfully oversees the FFE team’s product marketing, engineering and design and insists upon established rules to generate incremental results [9,52,53] because uncertainties that emerge along the road to a final commercialized product may divert the FFE team from the right direction.

As the post-COVID-19 era is making the future of the business world unpredictable, we believe that startup CEOs should focus on the presence of FFE through introspective mindfulness instead of worrying about the future. Mindfulness is a present-focused psychological practice intended to grow one’s spirituality that is deeply rooted in the Chinese Zen Buddhist meditation tradition [54]; the practice focuses on looking after one’s inner world mentally and eventually reaching inner peace. Hanh [55] explained mindfulness practices as follows: “The first step is awareness of the object, and the second step is looking deeply at the object to shed light on it” (p. 117), which involves trusting one’s own internal gut feelings. Therefore, CEOs provide firmness from their inner intuitive visions that they create through inner mindfulness practices.

## 3. Hypothesis development

### 3.1. FFE opportunity recognition and startup growth performance

To form an original, sound product concept, a manufacturing startup must observe business prospects in the market to identify available promising opportunities that can create FFE knowledge inflows for FFE innovation. Thus, we propose that the action of opportunity recognition is a key FFE activity for startups. The findings of Khurana and Rosenthal [8], Koen et al. [21], Kim and Wilemon [9] and Sandmeier et al. [43] all highlighted the importance of opportunity recognition as a crucial FFE activity, as opportunity recognition is a significant element that positively influences firms’ overall performance [56,57]. Ardichvili and Cardozo [58] also showed that opportunity recognition is a discovery process rather than a purposeful search and that this act requires entrepreneurial awareness. Therefore, there will certainly be obstacles in terms of unenforceable recognized ideas during the process of FFE. However, a substantial inflow of recognized opportunities provides more alternatives for better execution in later FFE activities based on Sandmeier et al.’s [43] funnel model. Moreover, Kuckertz et al. [16] revealed that opportunity recognition usually consists of high-intensity external knowledge searching; thus, searching for knowledge from the external market is vital in terms of a firm’s FFE opportunity recognition in relation to connecting seemingly irrelevant factors that are actually related to product concepts [59]. Therefore, FFE opportunity recognition is actually an intentional, active searching activity that requires an open environment. Consequently, we propose the following:

H1 FFE opportunity recognition positively impacts startup growth performance.

### 3.2. FFE concept generation and startup growth performance

The process of generating a product concept through FFE innovation is an effective way to decrease uncertainties and construct a primary market incentive concept by absorbing opportunities and ideas recognized externally while internally maintaining existing firm resources [33,60]. Therefore, FFE concept generation should be conducted alongside FFE opportunity recognition. We believe that FFE concept generation is the important action of identifying the most original product concept through an extensive idea selection and screening process based on opportunities recognized in the external market. Idea selection is the first step to shaping a concept depending on the funnel FFE model [43], where a final market-approved product concept has to start with the recognition of many possibilities to ensure precise selection [9]. All the selected ideas need to go through a screening process to identify the final feasible product concept. Consequently, we believe that FFE idea selection and FFE concept screening are the two main components of the FFE concept definition of this study [8,12,21,46,47]. Nobelius and Trygg [47] also pointed out the importance of concept screening in the context of executing FFE concept generation because FFE concept generation is a very time-consuming FFE activity and because firms need to carefully nurture product concepts through multiple procedures. All this evidence suggests that FFE product concept generation is vital in determining startup commercial success. Moreover, according to the findings of Eling et al. [61], the fastest way to generate a concept is to first select and screening ideas using intuition and then make a final product concept decision rationally with the help of concept screening; this is consistent with the fact that adopting two opposite mindsets enhances a startup’s innovation ability [1]. A careful concept screening procedure is used to weed out unpractical ideas that seem useful and retain good ideas that are mistakenly rejected [9]. Hence, we propose the following hypothesis:

H2 FFE concept generation positively impacts startup growth performance.

### 3.3. FFE vertical external involvement and startup growth performance

Urbinati et al. [62] emphasized the importance of external connections, including external stakeholders, for focal firms. An open cocreation FFE environment boosts a firm’s understanding of the market since interfirm connections not only improve a firm’s innovation ability but also drive a firm’s logistics, sales and marketing tactics forward [63–65]. Therefore, startups must apply an open innovation approach to expand external relationships with different external stakeholders during FFE innovation to deal with the market and supply chain uncertainties brought by the COVID-19 pandemic and realize external innovation resolution while initiating other FFE activities [13,14,23]. Huang et al. [48] showed that customers and suppliers are the two most important external FFE stakeholders since they share the core values of a manufacturing startup’s vertical value chain in terms of sustainable returns and social responsibility [66].

Alam [67] also showed that customer interaction reduces FFE uncertainty; Fang [68] revealed that the participation of customers accelerates the speed of product development; Tran et al. [51] creatively proposed that FFE customer involvement also helps elevate a final product’s aesthetic by integrating customer experiences to enable cocreation. Furthermore, under the downstream market-oriented perspective, customer involvement also helps firms increase their marketing, product delivery and customer service performance [69–70]. On the other hand, good, long-term, trustworthy collaboration between suppliers and focal firms promotes product development efficiency and has a positive influence on focal firms’ long-term growth [71]. Based on a study of 176 Chinese manufacturing firms, Feng et al. [72] showed that supplier involvement improves firms’ competitive advantage by decreasing their factory costs. Suppliers assist focal firms in meeting customers’ preferences by conducting market exploration and by offering experience with other focal firms, improving focal firms’ new product market acceptance based on potential market demand [19,73]. In summary, the FFE vertical external involvement of customers and suppliers brings external knowledge, markets demands and improvements of marketing strategies. Thus, we hypothesize the following:

H3a FFE customer involvement positively impacts startup growth performance.
H3b FFE supplier involvement positively impacts startup growth performance.

### 3.4. FFE prototyping and startup growth performance

The three preceding FFE activities are all conceptual; they do not involve creating an actual object. However, an FFE prototype is the first material object created in this process, and such prototypes embody products in a touchable way and represent the end of FFE innovation. Therefore, it is equally important to execute FFE prototyping since FFE prototyping has been used by firms to spur innovation and reduce uncertainty [12,74]. Christiansen and Gasparin [75] claimed that FFE prototyping provides alternative options rather than testing existing concepts through product experiments, adding more stability when a product is offered in the market.

Christiansen and Gasparin [28] pointed out the importance of creating a final product prototype during FFE innovation by combining human and nonhuman factors. Therefore, we believe that our open innovation model of FFE innovation increases disciplinary knowledge inflows to enhance FFE prototyping quality through the three other FFE activities. FFE prototyping can be seen as an open innovative bricolage process used to integrate different product opportunities, ideas and concepts into a practical model [76]; it is also a way for different stakeholders to preview a newly made product and inspect it from the perspectives of product creativity, feasibility and quality for future downstream commercialization phases [77]. In our opinion, FFE prototyping should be executed concurrently with the three other FFE activities to provide alternatives and correct product practical deficiencies to create a final polished prototype that can be considered for the production line; it is also an experimental procedure used to test the three other downstream market-oriented activities’ effectiveness during FFE that provides security during the product commercialization phase. Therefore, FFE prototyping helps everyone involved in the FFE process, improving firms’ commercial performance and thus promoting startups’ growth performance by reducing uncertainties and mistakes. Therefore, we hypothesize the following:

H4 FFE prototyping positively impacts startup growth performance.

### 3.5. The moderating effect of CEO mindfulness

Studies have proven that mindfulness practices can help entrepreneurs with stress reduction, resilience, work engagement, reduced turnover intentions, workplace relationships and communication, and overall firm performance [78,79], enhancing their decision-making mindset. Therefore, mindfulness practices are pivotal in terms of conscious self-attention and decision making when startup CEOs are leading firms through the uncertain COVID-19 pandemic era to realize expected growth [80]. FFE activities are usually characterized by strict business logic and commercial flexibility. A CEO’s mindfulness practices provide stable, soft power to encourage the FFE process. During the FFE process, CEOs’ mindfulness helps firms discover opportunities that bring them more growth benefits in ambiguous and complex markets [81]. It helps CEOs open their minds to the market and be aware of different business contexts.

Consequently, entrepreneurial mindfulness practices help startup CEOs capture diverse opportunities for decision-making in rapidly changing environments that are full of uncertainty [82,83], thus helping startups gain sustainable benefits. Mindfulness also helps entrepreneurs adopt an unbiased mindset in market analyses, which leads to a higher awareness for such entrepreneurs during highly precise evaluations and screening to generate appropriate product concepts [84,85]. A startup FFE team can generate more creative product concepts for product market commercialization if their CEO completely trusts the team based on his or her own intuition due to mindfulness practices, as FFE concept generation consists of both rational and intuitive approaches [86]. In addition to enhancing creativity during FFE, CEOs can increase the degree of involvement of customers and suppliers by concentrating on their own minds while leaving FFE activities to the team; in turn, this helps increase the FFE vertical involvement of customers and suppliers and thus increases the firm’s business performance. Gelderen et al. [87] emphasized that a high level of mindfulness spurs entrepreneurial action, which increases the degree of intensity of FFE prototyping. Moreover, as FFE prototyping provides alternative solutions for product concepts, Gordon and Schaller [84] proposed that mindfulness allows entrepreneurs to be flexible and aware of multiple perspectives instead of focusing on a single opinion, adding diverse dimensions to FFE prototype construction and thus increasing the probability of firm-level success. Hence, we hypothesize the following:

H5a CEOs’ mindfulness practices positively moderate the relationship between FFE opportunity recognition and startup growth performance.
H5b CEOs’ mindfulness practices positively moderate the relationship between FFE concept generation and startup growth performance.
H5c CEOs’ mindfulness practices positively moderate the relationship between FFE customer involvement and startup growth performance.
H5d CEOs’ mindfulness practices positively moderate the relationship between FFE supplier involvement and startup growth performance.
H5e CEOs’ mindfulness practices positively moderate the relationship between FFE prototyping and startup growth performance.

**Fig 2:**
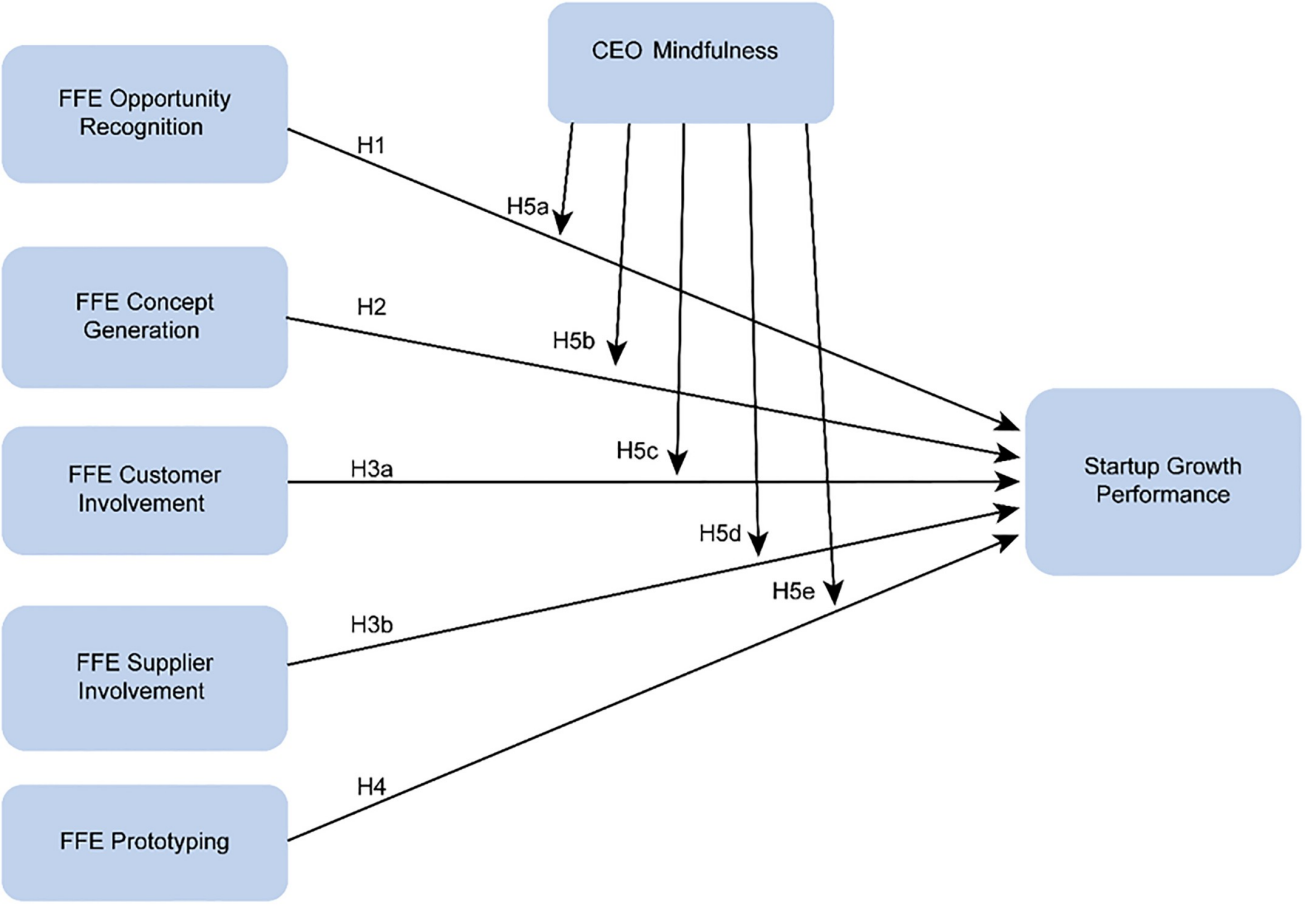
Research model.

In summary, on the basis of open innovation theory and from a market perspective, we construct a new model of the relationship between FFE activities and startup growth performance in this research (Fig 2). This study also examines the moderating effect of startup CEOs’ mindfulness on the relationship between market-oriented FFE activities and startup growth performance. We illustrate the relationship between FFE activities and growth performance by using an ancient Chinese philosophical perspective. We also provide insights into a startup CEO’s role in startup success.

## 4. Methodology

### 4.1. Survey design and sample

We adopted a quantitative, survey-based research design by using the structural equation modeling (SEM) method to investigate our research questions. We surveyed the CEOs of Chinese manufacturing startups that had implemented a formal FFE process [11] for three reasons. (1) During the post pandemic era, trade disputes and regional conflicts have introduced VUCA characteristics to global value chains, supply and demand needs and Chinese manufacturers’ innovation ability. Therefore, it is essential to investigate how these manufacturing companies have reacted to this VUCA era in terms of reintegrating existing resources for innovation. FFE is the initial phase of a manufacturer’s product innovation process. We hope to glean useful insights from our study on how Chinese manufacturers’ FFE activities increase market growth potential and thus provide valuable information to the global manufacturing industry. (2) As the VUCA cloud has been looming, business executives’ leadership must be impacted by all kinds of uncertainties. Thus, we also attempted to provide a solution from the perspective of a startup CEO, using CEOs’ mindfulness practices as a driver of startup manufacturers’ growth potential. As CEOs are often involved with their firms’ strategic plans [88,89], we hope our findings will show how CEOs’ ways of thinking can steer companies in the right direction during this VUCA age. (3) We integrated ancient Chinese philosophical elements into our research theory; therefore, our research is original in that we conduct practical research on how Chinese firms operate in the Chinese cultural environment from a global open innovation perspective.

The survey conducted in this study was an online questionnaire. First, investigating through the internet and universities partnered with startups, we researched Chinese manufacturing startups that had been established for less than 10 years, targeting those that had implemented a formal FFE procedure by inquiring through calls and emails. Second, we surveyed 70 manufacturing CEOs using a pilot field research survey to make minor improvements for additional precision, thus ensuring that our research questions were objective and rigorous. Third, we sent out the questionnaires to the CEOs and collected them via a professional Chinese survey platform, Survey Star (https://www.wjx.cn/). Fourth, we distributed the questionnaires to manufacturer CEOs through our research team’s personal networks and received 478 responses and 343 valid responses for a valid response rate of 72%. Finally, we used IBM SPSS 23.0 and IBM SPSS Amos 23.0 to analyze the sample data. The descriptive statistics of the sample are presented in Table 1. The questionnaires were sent out to obtain data on designated variables. The research hypotheses were also tested based on the collected data, and most of the items of the questionnaire were designed based on a 5-point Likert scale ranging from 1, representing “strongly disagree”, to 5, representing “strongly agree”. The survey questions were designed based on 343 different manufacturing startups’ performance in China over the past two years (2021–2022) when the COVID-19 pandemic was the most severe. The data sample covered different manufacturing sectors because we wanted to present a comprehensive industrial background to provide well-rounded evidence. The demographic map of different startup locations included China’s Shaanxi Province, Sichuan Province, Chongqing Municipality, Henan Province and Shandong Province, covering western China and the middle and lower reaches of the Yellow River area, where the startup economy has been promisingly increasing [90,91].

**Table 1:** Demographic characteristics of the sample.

Variables	Frequency	%
CEO company tenure		
Less than 3	140	40.8
3–5	110	32.1
6–10	93	27.1
CEO age		
Under 30	50	14.6
30–40	162	47.2
Over 40	131	38.2
CEO gender		
Male	320	93.3
Female	23	6.7
Firm age		
Less than 3	101	29.4
3–5	148	43.1
6–10	94	27.4
Firm size		
Fewer than 30	153	44.6
30–50	82	23.9
51–100	108	31.5
Average annual sales		
Less than 3 million	115	33.5
3–5 million	78	22.7
6–10 million	150	43.7
Industrial category		
Mechanical equipment manufacturing	53	15.5
Bio-chemical manufacturing	32	9.3
Electronic equipment manufacturing	3	0.9
Instrument manufacturing	26	7.6
Daily commodity manufacturing	213	62.1
Others	16	4.7

### 4.2. Measures

#### 4.2.1. Market-oriented FFE activities

We constructed five variables based on our theoretical summary of market-oriented FFE activities, listing FFE opportunity recognition, FFE concept generation, FFE customer involvement, FFE supplier involvement and FFE prototyping as our main independent variables (Table 2). The scales of both Laursen and Salter [92] and Hu and Zhang [93] concerning FFE opportunity recognition include recognizing opportunities from universities and research institutions. However, as our FFE opportunity recognition construct was market oriented, we consulted 70 manufacturing CEOs from our field research survey about useful opportunity recognition sources, and we drew inspiration from the research of O’Brien [94]. We finally narrowed down the outside market sources sought by FFE teams to three dimensions: industrial markets, government agencies and investors. Therefore, we created three items for the construct of FFE opportunity recognition, focusing on the aspect of capturing external opportunities based on the scale developed by Guo et al. [95]. According to previous studies, a statistical measurement scale for FFE concept generation has not been developed. However, both Li et al. [96] and Seboni [97] emphasized the market perspective in generating an original FFE concept in terms of brand building and concept novelty. Li et al. [96] and Riel et al. [98] added that the sustainability concerns of all the stakeholders of manufacturers are equally important in terms of generating a product concept. The three above facets of FFE concept generation were confirmed by the 70 manufacturing startup CEOs who participated in our field research survey interviews. Thus, we created three items that covered product brand attractiveness, product novelty and product sustainability development to form an FFE concept generation construct. FFE vertical external involvement functions as an important liaison enabling FFE teams to stay connected with market influences. Therefore, we adopted the approach of Menguc et al. [50], creating three items each for FFE customer involvement and FFE supplier involvement to examine the connectiveness between startups and their vertical external stakeholders. As is the case with FFE concept generation, there is no mature FFE prototyping measurement scale in previous scholarly literature; instead, FFE prototyping is a material building-based activity. Therefore, we focused on the quality, cost and manufacturing ability of FFE prototypes based on the insights of Li et al. [96] and Seboni [97], creating three items to shape the FFE prototyping construct.

**Table 2:** Report of reliability and validity.

Constructs	Indicators	Factor loading	CR	AVE	Cronbach’s α
FFE opportunity recognition	We frequently identify opportunities from industrial markets, government agencies and investors to launch new products during FFE innovation	0.661	0.751	0.510	0.846
	We frequently identify ideas from industrial markets, government agencies and investors that can be converted into new products during FFE innovation	0.738			
	We enjoy thinking about new ways to develop products during FFE innovation	0.723			
FFE concept generation	We seriously consider products’ brand attractiveness in their original conceptual form during FFE innovation	0.847	0.866	0.683	0.862
	We seriously consider products’ novelty when forming original concepts during FFE innovation	0.859			
	We seriously consider products’ sustainability when forming original concepts during FFE innovation	0.770			
FFE customer involvement	We always consider product reviews from customers during FFE innovation	0.847	0.896	0.741	0.853
	We always establish pilot tryouts with selected customers during FFE innovation	0.895			
	Our FFE team is cross functional with customers	0.839			
FFE supplier involvement	We always consider product reviews from suppliers during FFE innovation	0.847	0.900	0.749	0.885
	Our FFE team is cross functional with suppliers	0.893			
	We always share FFE plans with suppliers	0.856			
FFE prototyping	We seriously verify products’ quality when fabricating product prototypes during FFE innovation	0.830	0.850	0.654	0.837
	We seriously verify products’ manufacturing costs when fabricating product prototypes during FFE innovation	0.821			
	We seriously verify products’ manufacturability when fabricating product prototypes during FFE innovation	0.773			
CEO’s mindfulness	My mood does not change when the business environment is turbulent	0.710	0.866	0.566	0.735
	I always focus on the present rather than paying attention to the past or future	0.833			
	It is easy for me to pay attention to my inner thoughts	0.787			
	I have a strong ability to deal with pressure	0.705			
	It is easy for me to face setbacks or failures	0.717			
Startup growth performance	The startup’s ROI has increased over the past two years more than that of other firms in the industry	0.822	0.840	0.637	0.823
	The startup’s ROS has increased over the past two years more than that of other firms in the industry	0.841			
	The startup’s market share has grown over the past two years more than that of other firms in the industry	0.727			

#### 4.2.2. CEO mindfulness

We measured CEO mindfulness by reforming the mindful attention awareness scale (MAAS) developed by Brown and Ryan [99]. The MAAS is the most broadly used and frequently cited mindfulness measure that can be applied to both clinical and nonclinical studies [100–102]. We integrated the essence of the MAAS by applying it to our research. We reduced the number of items in the MAAS from 15 to 5 to adapt it to a business context (Table 2). We embedded our 5-item mindfulness scale to five aspects related to CEOs’ psychological condition: the degree of mood changes, the degree of focus on the present, the degree of attention given to one’s own inner world, the degree of resilience to pressure and the degree of tenacity.

#### 4.2.3. Startup growth performance

We used three measures to capture startup growth performance, adopting three developed subjective items from previous studies with the same effect as an objective scale [103,104]. These three aspects of growth correspond to return on investment (ROI) growth, return on sales (ROS) growth and market share growth over the last two years compared with those of other companies in the industry [105].

#### 4.2.4. control variables

Fim age, firm size, CEO age and CEO gender.

#### 4.2.5. Common method bias

The questionnaires were completed anonymously, and the respondents were informed in advance that there were no right or wrong answers to mitigate exaggerated cognitive errors. We also used two methods to test for common method bias (CMB) in our data. We first used Harman’s single-factor test. The first unrotated extracted factor loading was 29.42%, showing that there was no significant CMB. Second, we applied the latent variable approach. We loaded all the items on their constructs and on a latent CMB factor. Next, we examined the significance of the constructs both with and without the latent factor. The results showed that all the relationships are consistently significant, indicating that CMB is not an issue in this research.

## 5. Analysis and results

We used the SEM method in this study for analysis and calculation since most studies that contain psychological variables use SEM method. First, we used IBM SPSS 23 to test the reliability and validity of our sample data. Next, we employed a traditional method to assess our hypotheses using IBM SPSS Amos 23.

### 5.1. Reliability and validity

Table 2 shows the results of the comparative factor analysis. All the constructs’ Cronbach’s alpha values are greater than 0.70, and all their CR values are greater than 0.70, indicating good reliability. The results also show that the sample data have good internal consistency. All the items converge to seven constructs according to our theoretical model. Each item’s factor loading is greater than 0.6, which indicates that all the items are statistically significant. This indicates that the data sample is acceptable for subsequent analysis. The results of the KMO measure and Bartlett’s test of sphericity are both satisfactory. The KMO = 0.832, which is greater than 0.7. The approximate chi square of Bartlett’s test of sphericity is 4494.555, and p = 0.000. At the construct level, we examined the convergent validity of the constructs via their average variance extracted (AVE) values (Table 2). All the constructs’ AVE values are greater than 0.5, indicating good convergent validity. Our questionnaire also went through a pilot survey for minor corrections; therefore, the content validity is also good. We further tested the correlations between different constructs and found correlations between each pair of constructs, providing statistical support for subsequent analysis (Table 3). Table 3 also shows that the square root of each construct’s AVE value is greater than the correlation coefficient of that construct and each of the other constructs, indicating good discriminant validity.

**Table 3:** Inter-construct correlations.

**Variables**	**1**	**2**	**3**	**4**	**5**	**6**	**7**
1. FFE opportunity recognition	1						
2. FFE concept generation	0.528**	1					
3. FFE customer involvement	0.239**	0.157**	1				
4. FFE supplier involvement	0.345**	0.317**	0.177**	1			
5. FFE prototyping	0.380**	0.353**	0.289**	0.318**	1		
6. CEO mindfulness	0.498**	0.452**	0.149**	0.288**	0.349**	1	
7. Startup growth performance	0.441**	0.402**	0.257**	0.338**	0.423**	0.398**	1
Mean	3.703	4.144	3.982	3.903	4.178	3.987	3.842
SD	0.931	0.837	0.870	0.843	0.729	0.760	0.913

** < 1%.

### 5.2. Hypothesis testing

According to the results of the goodness-of-fit test we conducted using IBM SPSS Amos 23, the values are all within the recommended ranges (Table 4), which indicates that the structural equation model is structurally valid. We then conducted hypothesis testing, using IBM SPSS Amos 23 to test the positive and significant relationships between FFE activities and startup growth performance (Table 5). Our analytical results showed that even though the relationship between FFE opportunity recognition and startup growth performance is positive, the relationship is nonsignificant (b = 0.193, n.s.), rejecting H1. However, the relationship between FFE concept generation and startup growth performance is positive and significant (b = 0.256, p < 0.01), supporting H2. We also examined the relationship between FFE vertical external involvement and startup growth performance. The results showed that there is no significant relationship between FFE customer involvement and startup growth performance (b = 0.129, n.s.), rejecting H3a. However, the relationship between FFE supplier involvement and startup growth performance was shown to be significant (b = 0.195, p < 0.01). Finally, our results illustrated that the relationship between FFE prototyping and startup growth performance is significant at the 0.001 level (b = 0.341, p < 0.001).

**Table 4:** Goodness fit simulation report.

Category	χ2	χ2/df	CFI	GFI	NFI	IFI	RMSEA
Values	328.803	2.740	0.942	0.940	0.913	0.943	0.054
Recommended values	> 0	< 3	> 0.9	> 0.9	> 0.9	> 0.9	< 0.06

**Table 5:** Report of hypothesis testing between FFE activities and startup growth performance.

Hypotheses	Path coefficient	C.R	S.E	P value
H1	0.193	0.593	0.074	n.s.
H2	0.256	2.760	0.093	**
H3a	0.129	0.945	0.066	n.s.
H3b	0.195	2.663	0.073	**
H4	0.341	5.916	0.087	***

* < 5%,

** < 1%.

Next, we examined the moderating effect of CEO mindfulness effects on the relationship between FFE activities and startup growth performance using IBM SPSS Amos 23 (Table 6). We first constructed five interactions with each of the five FFE constructs. The interaction between FFE opportunity recognition and startup growth performance was shown to be positive and significant (b = 0.345, p <0.05); hence, H5a was supported. The interaction between FFE concept generation and startup growth performance, as well as that between FFE customer involvement and startup growth performance, was shown to be positive and significant (b = 0.089, p < 0.01; b = 0.147, p < 0.01). Therefore, H5b and H5c are both supported. However, H5d and H5e are both rejected due to nonsignificant p values (b = 0.233, n.s.; b = 0.171, n.s.). Hence, we rejected H5d and H5e.

**Table 6:** Report of the moderating effects of CEO mindfulness.

Hypotheses	Path coefficient	C.R	S.E	P value
H5a	0.345	4.172	0.072	*
H5b	0.089	1.432	0.13	**
H5c	0.147	2.113	0.068	**
H5d	0.233	0.703	0.083	n.s.
H5e	0.171	0.654	0.084	n.s.

* < 5%,

** < 1%.

### 5.3. Robustness test

In order to secure the robustness of the moderating effects, this research utilized Bootstrap method to conduct robustness test of CEO mindfulness’s moderating effects by using IBM SPSS 23. During the Bootstrap test, we set sample size as 5000, confidence interval as 95%. The results are shown in Table 7.

**Table 7:** Robustness test of moderating effects.

Path	Moderating effect of CEO mindfulness	Effect value	95% confidence interval	
			Boot CI minimum	Boot CI maximum
FFE opportunity recognition→Startup growth performance	Low (M-1SD)	0.264	0.148	0.280
	Medium (M)	0.370	0.267	0.441
	High (M+1SD)	0.476	0.365	0.586
FFE concept generation→Startup growth performance	Low (M-1SD)	0.184	0.071	0.297
	Medium (M)	0.294	0.214	0.373
	High (M+1SD)	0.403	0.297	0.509
FFE customer involvement→Startup growth performance	Low (M-1SD)	0.238	0.116	0.361
	Medium (M)	0.376	0.286	0.467
	High (M+1SD)	0.514	0.384	0.645
FFE supplier involvement→Startup growth performance	Low (M-1SD)	0.093	-0.401	0.185
	Medium (M)	0.096	-0.439	0.274
	High (M+1SD)	0.120	-0.532	0.308
FFE prototyping→Startup growth performance	Low (M-1SD)	0.253	-0.151	0.354
	Medium (M)	0.331	-0.254	0.409
	High (M+1SD)	0.410	-0.298	0.522

As Table 7 shows, except CEO mindfulness’ moderating effects on the relationship between FFE supplier involvement—startup growth performance and FFE prototyping—startup growth performance, all other 95% confidence intervals of CEO mindfulness’ moderating effects do not contain 0, which corresponds to the hypothesis tests. Therefore, we confidently prove that CEO mindfulness positively moderates the relationship between FFE opportunity recognition—startup growth performance, FFE concept generation—startup growth performance and FFE customer involvement—startup growth performance.

## 6. Conclusions

### 6.1. Discussion

Our findings rejected H1, showing a nonsignificant relationship between FFE opportunity recognition and startup growth performance. We believe this is because the opportunities change over time within the COVID-19 VUCA business environment; thus, product opportunities become ineffective when products are finally on the market for sale since business opportunities change rapidly under the rapidly changing COVID-19 market conditions. On the other hand, intensive opportunity searches are time consuming, decreasing focus on polishing specific products. However, our findings supported H2, showing that FFE concept generation has a positive effect on startup growth performance. As FFE concept generation includes idea selection and idea screening, this FFE activity specifically generates valuable product concepts that provide security against omitting good ideas and eliminates ideas that are unfeasible for final market use precisely [9,106,107]. Third, our statistical results rejected the hypothesis that FFE customer involvement has a positive effect on startup performance but supported the significant relationship between FFE supplier involvement and startup performance through FFE vertical external involvement, rejecting H3a and supporting H3b. This proves that taking customers’ advice and preferences seriously during the FFE phase may lead to customer bias, resulting in market shortsightedness and harming product innovation [108,109]. However, in contrast with customers, suppliers are usually tied with manufacturers for a common economic goal, and they should know the market operation well and thus be able to provide useful knowledge. This shows the importance of the supply chain. Finally, our findings strongly supported H4. FFE prototyping is an affirmative activity that tests the practicality of product concepts. This approach is essential to the later stages of the NPD process when a product is undergoing a massive production process. Hence, it determines startups’ commercial success.

However, due to the COVID-19 global pandemic, it has become clear that firms’ CEOs need to have strong spirituality to make appropriate decisions that guide firms out of crises via FFE innovation. The condition especially applies to startup companies since they are often small and financially fragile. Hence, we further examine the moderating effects of CEO mindfulness on the investigated relationships, as mindfulness practices can help CEOs make appropriate decisions under extreme pressure to derive future benefits [22]. First, our findings supported H5a, H5b and H5c, indicating that CEO mindfulness positively moderates the relationships between FFE opportunity recognition, FFE concept generation, FFE customer involvement and startup growth performance. Gelderen et al. [87] proposed that CEO mindfulness elevates entrepreneurial task performance. A substantial level of FFE opportunity recognition intensity sometimes confuses startup FFE teams with different options, and CEO mindfulness points these teams in the right direction in terms of the commercial success of products. Both FFE concept generation and FFE customer involvement need appropriate high-level decision making to produce suitable concepts and integrate customer advice. However, suppliers are generally more experienced in their own decision making, thus providing valuable advice without supervision during the FFE process. In contrast, FFE prototyping is a rational step-by-step activity that requires implementation capacity instead of intuitive top management orders. Hence, H5d and H5e are rejected.

### 6.2. Theoretical implications

We creatively combined Chinese Taoism Yin-Yang thought with our market-oriented FFE open innovation model in this study, adopting an ancient Chinese harmonious approach [24,25]. Through logical reasoning and empirical analysis, it has proved that Chinese Yin-Yang approach can increase manufacturing startups’ growth performance in the long run. We also creatively categorized FFE activities based on market commercialization. This FFE model connects market-related FFE activities and startup growth performance in an entrepreneurial way, and most FFE activities have positive impacts on startups’ growth performance. We further tested the moderating effect of startup CEO mindfulness practices. We have made three major theoretical contributions through this research. First, we constructed an FFE open innovation model that addresses FFE activities and FFE uncertainties in this COVID-19-affected VUCA age. Unlike previous scholars, we believe that the influence of uncertainties is an accelerating mechanism for FFE innovation, provoking startups to engage in more innovation and achieve greater commercial success. FFE activities and FFE uncertainties influence each other, so they ultimately reach an innovation equilibrium. Second, we examined the relationships between FFE activities and startup growth performance from a downstream market commercialization perspective based on our open innovation FFE model, taking external market considerations into account in the context of the implementation of FFE activities will increase startup growth performance. We believe that it is theoretically important to consider marketing elements in advance during the FFE phase to enable greater economic success in an open innovation environment. We also found that FFE concept generation, FFE supplier involvement and FFE prototyping have positive impacts on startup growth performance. Finally, we examined the moderating effects of startup CEO mindfulness, which originated from ancient Chinese Zen tradition, on the relationship between FFE activities and startup growth performance from a micro perspective. We found that CEO mindfulness positively moderates the relationships between FFE opportunity recognition and startup growth performance, FFE concept generation and startup growth performance and FFE customer involvement and startup growth performance. Our findings supported the equal importance of both human and nonhuman players in the context of FFE innovation [28].

### 6.3. Managerial implications

It is generally believed that FFE innovation is a fiercely confidential business process because it addresses a manufacturing company’s initial core strategies in terms of new product growth performance. However, our study reveals that acquiring different resources and knowledge from both inside and outside a firm generate unexpected outcomes. Furthermore, our study also indicates that FFE innovation determines a product’s later commercial success. We made three main managerial implications in this study.

First, it is essential to open up startup companies’ boundaries during FFE innovation to connect with different knowledgeable resources that can benefit both internal and external stakeholders. All internal and external players can help build a firm’s knowledge base, enabling it to better execute FFE activities in an open innovation environment. The second law of thermodynamics indicates that an isolated system will only exhibit increasing entropy and ultimately chaos, which illustrates that any physical organization should be opened up to its external environment to reduce chaotic uncertainties. External stakeholders such as suppliers provide vital knowledge during FFE innovation for startup companies’ long-term economic growth; second, the priority of FFE activities should be market-centralized strategy rather than technology-focused strategy. Startup companies should first identify a market trend during FFE innovation and then work on technology R&D to meet market demands. These market-oriented FFE activities should also be carried out in a concurrent manner to realize full innovation potential. However, as startup companies are newer to their industries than other mature firms, startup companies should not overestimate the importance of FFE opportunity recognition and FFE customer involvement because opportunities and customer preferences change quickly over time. Steve Jobs also once said that customers do not truly know what they want. Therefore, it is relatively important for startups to execute concurrent FFE activities with a focus on holistic market trends and demands. Third, ancient Chinese philosophical wisdom raises the dimensionality of FFE innovation practices to a new level, enabling survival of the COVID-19 pandemic. Previous FFE practices are all based on a Western point of view, where FFE teams attempt to decrease FFE uncertainties as much as possible. However, our study shows that a Taoism Yin-Yang approach allows startups to treat uncertainties in FFE innovation as a routine, especially during this VUCA age. Conversely, FFE uncertainties increase startups’ FFE innovation ability, enabling them to survive the pandemic. At the same time, a CEO’s mindfulness practice-based leadership rooted in Chinese philosophy also helps make FFE activities more innovative and effective in a nonradical way. CEO mindfulness enhances the relationships between originality-related market-oriented FFE activities (FFE opportunity recognition, FFE concept generation and FFE customer involvement) and startup growth performance. Startup CEOs should remain calm and monitor their own inner peace to provide FFE innovation guidance when their external business environment is turbulent.

### 6.4. Limitations and future research

This study has several limitations that provide opportunities for future research. First, our study summarized market-focused FFE activities. However, as noted above, there should be technology-focused FFE activities. The mechanism of technology-focused FFE activities should be different from that of market-oriented FFE activities and is yet to be discovered. Second, as carbon neutrality and environmentally friendly actions have been a focus of many companies, it will be important to investigate the effect of green innovation on FFE innovation in the future. Third, this study focuses on only manufacturing startup FFE innovation. However, FFE innovation in the service industry represents an area for future academic exploration. Fourth, we have investigated only one psychological trait of startup CEOs. However, there should be other psychological traits of CEOs that affect FFE innovation.
